# DHA, a potentially therapeutic lipid for myocardial infarction

**DOI:** 10.1093/procel/pwaf073

**Published:** 2025-08-06

**Authors:** Chenglu Xiao, Jing-Wei Xiong

**Affiliations:** School of Basic Medical Sciences, The Second Affiliated Hospital, Institute of Biomedical Innovation, The MOE Basic Research and Innovation Center for the Targeted Therapeutics of Solid Tumors, Jiangxi Medical College, Nanchang University, Nanchang 330031, China; School of Basic Medical Sciences, The Second Affiliated Hospital, Institute of Biomedical Innovation, The MOE Basic Research and Innovation Center for the Targeted Therapeutics of Solid Tumors, Jiangxi Medical College, Nanchang University, Nanchang 330031, China

Myocardial infarction results in the loss of a massive amount of cardiomyocytes (CMs), ultimately leading to heart failure (HF). Although current medical therapeutics can alleviate the symptoms of myocardial infarction, they cannot eliminate the ischemia-induced CM loss (Hume et al., 2023). In adult mammals, the reparatory response to myocardial infarction is the formation of scar tissue, which compromises heart function (Ebrahimi, 2018). For decades, the adult heart has been regarded as a post-mitotic organ, with CMs typically characterized as terminally differentiated that are unable to proliferate (Hashmi and Ahmad, 2019). In contrast to adult mammals, adult zebrafish possess the full capacity of cardiac regeneration after ventricular resection (Poss et al., 2002). In the first 7 days after birth, the neonatal mouse heart can also fully regenerate. However, this regenerative ability is quickly lost after birth for 7 days (Porrello et al., 2011). In the regenerating hearts, CMs undergo dedifferentiation and proliferation to supply new CMs (Jopling et al., 2010; Kikuchi et al., 2010). Elucidating the mechanisms underlying CM proliferation and cardiac regeneration is of paramount significance. A wealth of research works has demonstrated a close correlation between CM proliferation and metabolism. The induction of metabolic reprogramming in CMs to promote cardiac regeneration has emerged as a cutting-edge and highly active area of research works within the field of cardiac regeneration.

Metabolic shifts from glycolysis to oxidative metabolism, which occur during development and some cardiac diseases, are thought to influence the capacity of CMs to reinitiate the cell cycle and undergo proliferation. The embryonic heart mainly utilizes glucose metabolism for energy production. After birth, in order to adapt to the environment and changes in growth pressure, the CMs undergo metabolic remodeling (Puente et al., 2014). During the immediate postnatal period, the primary energy source is derived from glycolysis. However, by the 7 days after birth, there is a notable decline in glycolytic activity, coinciding with an increase in fatty acid oxidation (FAO) metabolism. In the adult mammalian heart, myocardial infarction leads to metabolic reprogramming, but these changes are insufficient to stimulate CM proliferation to repair the lost CMs by damage (Chen et al., 2024).

Currently, numerous investigations have demonstrated that modifying metabolic processes can impact the proliferation of CMs. It has been well known that induction of hypoxia in adult animals results in inhibition of oxidative metabolism and reactivation of cardiomyocyte mitosis (Nakada et al., 2017). YAP may regulate metabolic genes for its ability of promoting cardiomyocyte proliferation (Wang et al., 2018). A study has also identified a cocktail of five small molecules that efficiently induced cardiomyocyte proliferation partially by turning on CM metabolic switching toward glycolysis/biosynthesis (Du et al., 2022). Other studies have shown that manipulating metabolic enzymes in metabolic pathways, especially glycolysis and FAO, is sufficient to induce CM proliferation, such as GLUT1 overexpression promotes neonatal heart regeneration (Fajardo et al., 2021); introducing modified mRNA of Pyruvate Kinase Muscle Isozyme 2 (PKM2) into the heart after myocardial infarction leads to an increase in cardiomyocyte proliferation; and HMGCS2 overexpression increases adult cardiomyocyte dedifferentiation and proliferation after heart injury (Cheng et al., 2022).

While the majority of studies demonstrate that inhibiting FAO can enhance cardiomyocyte proliferation, other investigations have drawn different conclusions. Carnitine palmitoyltransferase-1 (CPT1), normally located at the mitochondrial outer membrane, acts as a rate-limiting enzyme for mitochondrial β-oxidation by regulating the entry of long-chain fatty acids into the mitochondria. Among the three CPT1 isoforms (CPT1a, CPT1b, and CPT1c), CPT1b is the primary isoform in the adult heart and a key target for metabolic interventions aimed at enhancing cardiac function in patients with cardiac hypertrophy and HF through FAO inhibition (Brown et al., 1995; He et al., 2012). Small-scale clinical trials have shown that CPT1 inhibitors, like etomoxir, can benefit HF patients (Schmidt-Schweda and Holubarsch, 2000). However, animal studies (Table 1) have shown mixed results (He et al., 2012; Lionetti et al., 2005; Schwarzer et al., 2009). One study has found that cardiac-specific knock out of *Cpt1b* allows heart regeneration in adult mice after ischemia–reperfusion injury (Li et al., 2023). Despite uncovering the different molecular mechanisms, others have reached similar conclusions: cardiac-specific deletion of *Cpt1a* and *Cpt1b* enhanced cardiomyocyte proliferation and improved cardiac function post-MI (Tang et al., 2025). Interestingly, recent investigations have yielded contrasting results during zebrafish heart regeneration, revealing that the deletion of *cpt1b* induces lipid accumulation and substantially hampers ventricular regeneration (Zhao et al., 2024), and that knocking out the entire *cpt1b* coding sequence impairs cardiomyocyte proliferation, whereas CM-specific overexpression of *cpt1b* enhances it (Cheng et al., 2024). Notably, a recent work from Dr. Jun Chen’s lab, which is under consideration in *Protein & Cell*, revealed that knockout of *cpt1ab* leads to increased lipid accumulation in the injury area and enhances heart regeneration in zebrafish. This highlights the highly complex role of FAO in heart regeneration, which warrants further investigations on its regulatory mechanisms in cardiac development, diseases, and regeneration. The fact that cardiac-specific knockout of *Cpt1b* in mice did not induce lipotoxicity (Li et al., 2023) suggests that generating the zebrafish line with *cpt1*-specific knockout may offer additional clues regarding the regulatory mechanisms of FAO. Additionally, the functions of different CPT family members during cardiac regeneration and the conservation of their function across different species remain to be addressed in the future.

**Table 1.** CPT1 inhibition in different models reveals contradictory outcomes on CM proliferation and regeneration.

Targeted genes	Manipulations	Effects	References
*cpt1b*	Analyze the CM proliferation of *cpt1b*^−/−^ mutant or CM-specific *cpt1b* overexpression zebrafish hearts.	CM-specific overexpression of *cpt1b* induced CM proliferation.	(Cheng et al., 2024)
*cpt1b*	The *cpt1b*^−/−^ mutant zebrafish larvae were subjected to ventricle ablation.	Impeded zebrafish ventricle regeneration and CM proliferation.	(Zhao et al., 2024)
*Cpt1a* and *Cpt1b*	Cardiac-specific *Cpt1a* or *Cpt1b* knockout mice were subjected to MI; Etomoxir treated adult mice after MI.	Ameliorated the pathological effects of MI by inducing CM proliferation.	(Tang et al., 2025)
*Cpt1b*	Cardiac-specific *Cpt1b* knockout mice were subjected to ischemia–reperfusion (I–R) injury.	Enabled heart regeneration.	(Li et al., 2023)
*Cpt1b*	CPT1b knockout (CPT1b^+/−^) mice were subjected to transverse aorta constriction-induced pressure overload.	Caused lipotoxicity in the heart under pathological stress and exacerbated the cardiac pathological development.	(He et al., 2012)
*Cpt1*	Etomoxir and NVP-LAB121 treated pressure overload-induced HF in rats.	Failed to reverse HF.	(Schwarzer et al., 2009)
*CPT1*	Oxfenicine treated pacing-induced HF in dogs.	Prevented left ventricular wall thinning and delayed the time to end-stage failure.	(Lionetti et al., 2005)

Briefly, the recent work from Dr. Chen’s lab not only discovered the positive effect of lipid accumulation in zebrafish regeneration, but also found that docosahexaenoic acid (DHA) has a conserved essential role in promoting cardiac regeneration among different species. Their data revealed that DHA promotes adult zebrafish and neonatal mouse CM proliferation upon heart injury, and genes responsible for DHA synthesis were only activated in the injured heart of zebrafish and neonatal mice, not in the adult mice. Moreover, DHA supplementation promoted CM proliferation, inhibited inflammation and fibrosis in adult MI mouse heart through PPARD. While the DHA/PPARD signaling pathway may offer promise in treating HF, the precise dosage of DHA, the method of delivery, and safety considerations are crucial for future clinical studies.

## Data Availability

Not applicable.

## Abbreviations

CPT1: carnitine O-palmitoyltransferase 1
CPT: carnitine O-palmitoyltransferase
CM: cardiomyocyte
CMs: cardiomyocytes
DHA: docosahexaenoic acid
FAO: fatty acid oxidation
HF: heart failure
GLUT1: glucose transporter-1
HMGCS2: hydroxymethylglutaryl-CoA synthase 2
PKM2: Pyruvate Kinase Muscle Isozyme 2
PPAR: peroxisome proliferator-activated receptors
YAP: Yes-associated protein
