# Correction to: DHA, a potentially therapeutic lipid for myocardial infarction

**DOI:** 10.1093/procel/pwaf088

**Published:** 2025-12-03

This is a correction to: Chenglu Xiao, Jing-Wei Xiong, DHA, a potentially therapeutic lipid for myocardial infarction, *Protein & Cell*, 2025; https://doi.org/10.1093/procel/pwaf073

The following changes have been made to the originally published manuscript. An addition has been added to the **References** list: “Tang Z, Sun Z, Yang C *et al.* Accumulation of newly synthesized docosahexaenoic acid plays an essential role in heart regeneration. *Protein & Cell* 2025, 10.1093/procel/pwaf062.” Also, links to this work in the paper (“(Tang et al., 2025)”) have been updated throughout the paper.

